# Fractal Modelling of Heterogeneous Catalytic Materials and Processes

**DOI:** 10.3390/ma17215363

**Published:** 2024-11-01

**Authors:** Suleiman Mousa, Sean P. Rigby

**Affiliations:** 1 Department of Chemical Engineering, King Faisal University, P.O. Box 400, AlAhsa 31982, Saudi Arabia; saamousa@kfu.edu.sa; 2 Department of Chemical and Environmental Engineering, Faculty of Engineering, University of Nottingham, University Park, Nottingham NG7 2RD, UK

**Keywords:** fractal dimension, multifractal, pore network, catalyst support, forming, SAXS, X-ray tomography, FIB-SEM, MRI, computer simulation

## Abstract

This review considers the use of fractal concepts to improve the development, fabrication, and characterisation of catalytic materials and supports. First, the theory of fractals is discussed, as well as how it can be used to better describe often highly complex catalytic materials and enhance structural characterisation via a variety of different methods, including gas sorption, mercury porosimetry, NMR, and several imaging modalities. The review then surveys various synthesis and fabrication methods that can be used to create catalytic materials that are fractals or possess fractal character. It then goes on to consider how the fractal properties of catalysts affect their performance, especially their overall activity, selectivity for desired products, and resistance to deactivation. Finally, this review describes how the optimum fractal catalyst material for a given reaction system can be designed on a computer.

## 1. Introduction

**Figure 1:**
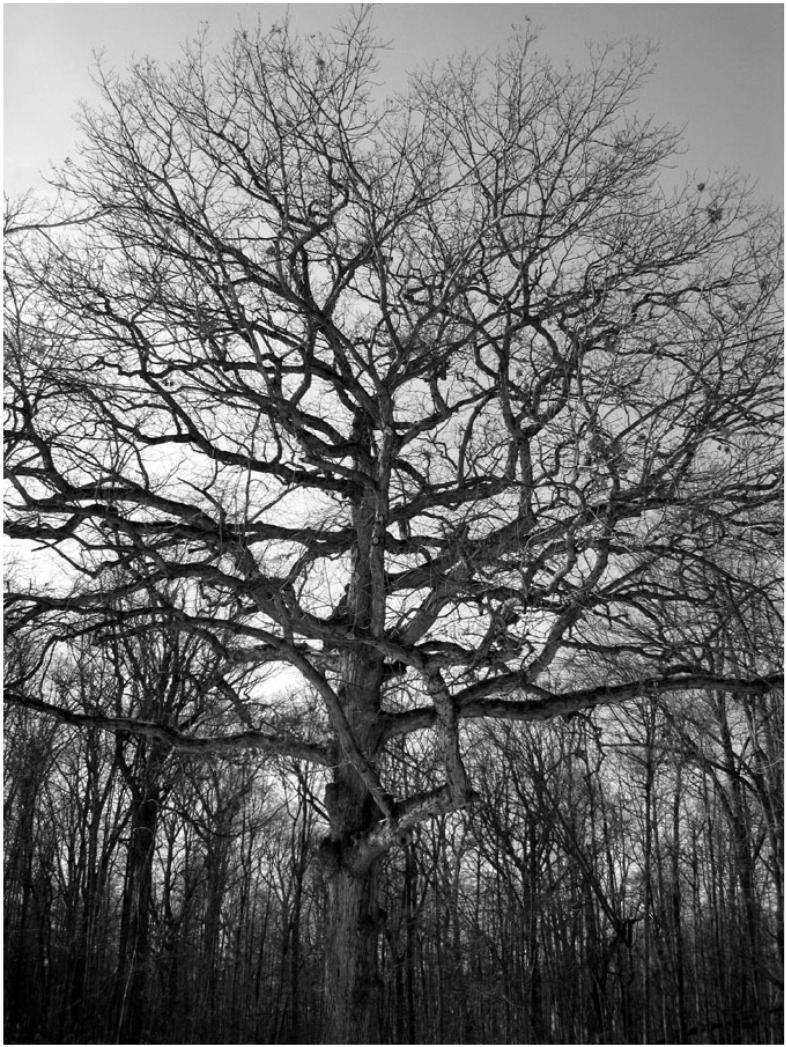
Fractal tree branches. Author: John Leszczynski. This image is licensed under the Creative Commons Attribution-Share Alike 2.0 Generic license.

Fractals arise frequently in Nature, such as the fractal tree structure shown in Figure 1. The fact that Nature has frequently, through the process of evolution, developed fractal-like structures for its own ‘bioreactors’, such as the tree-like structure of the tubes within lungs or the blood distribution network in kidneys, when trying to provide an optimal transport network, suggests that fractal structures may provide optimal designs for catalytic materials, especially for diffusion-limited reactions. For catalyst materials, the optimal design provides for the best catalytic activity, selectivity, and resistance to deactivation.

**Figure 2:**
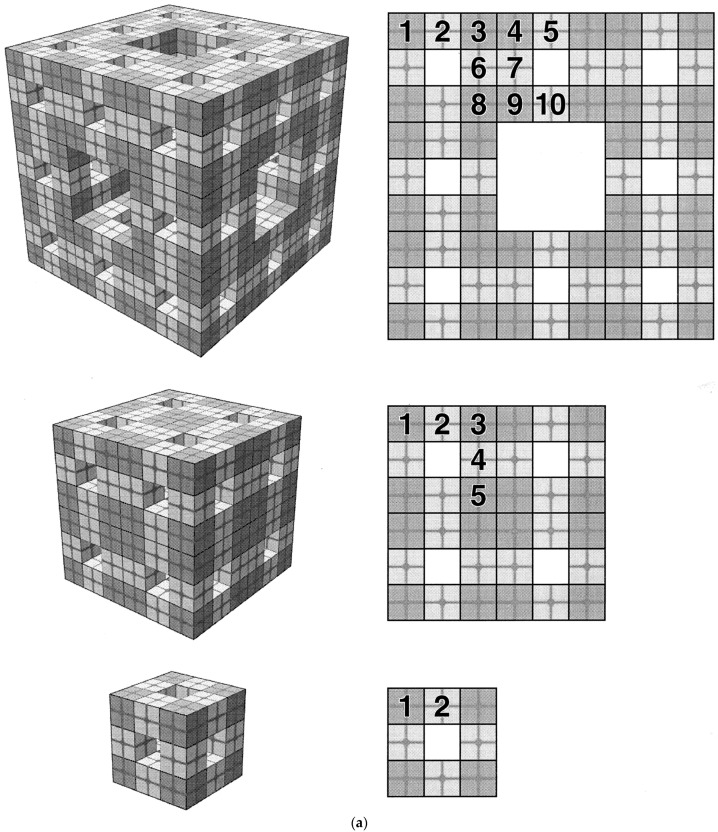

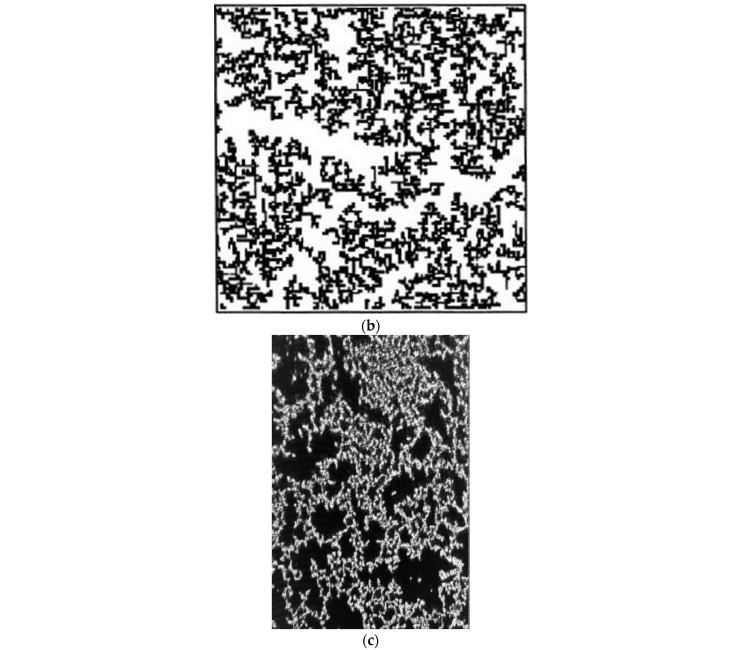
(**a**) Regular/exact 3D Menger sponge (pre-)fractal. Reprinted with permission from Ref. [2], 1999, American Chemical Society. The bottom, initial generator structure is used to produce the topmost, next level in the fractal creation process. This process can be repeated to ever-more levels. In contrast, the type of repetition in the middle structure is not fractal. (**b**) Computer-simulated 2D cluster–cluster aggregate (CCA) random (statistical) fractal model (black = CCA). Reprinted with permission from Ref. [3], 1996, Elsevier (**c**) An example of the natural occurrence of such a CCA-type fractal in the form of a precipitated silica film (white = silica, black = void). Reprinted with permission from Ref. [4], 2005, American Chemical Society.

Fractals are objects that possess the special property of self-similarity [1]. This property means that they possess a similar geometry that recurs at ever-smaller length scales. Closer inspection at ever-smaller length scales reveals repeated forms. This new sort of symmetry may be of an exact or statistical nature. For ideal, exact fractals, a simple generator structure is repeated at ever-smaller length scales. The natural tree shown in Figure 1 possesses some statistical self-similarity, since if a close-up image of part of it were presented without scale or context, it would be hard to discern from which generation of branches and/or twigs it had been taken. Examples of both exact and statistical fractal structures are given in Figure 2. An overall object that is some sort of composite that contains more than one type of fractal is sometimes called a “multi-fractal” [1].

**Figure 3:**
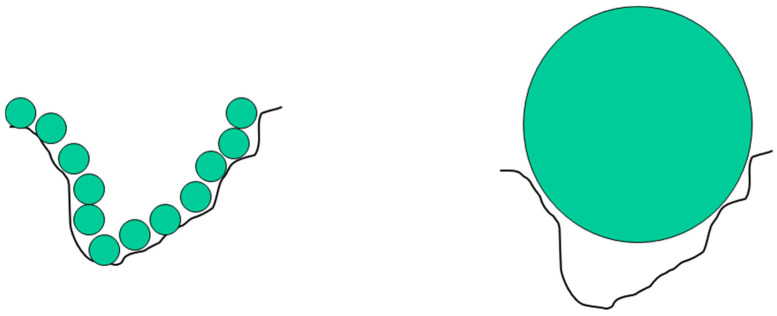
Schematic diagram illustrating the molecular sieving effect for rough surfaces depending upon molecular size. The smaller molecules can enter surface convolutions from which the larger molecule is excluded.

Another key feature of fractal objects is that measurements of characteristic parameters, such as surface area or pore volume, depend upon the size of the ‘ruler’ or ‘yardstick’ used to perform the measurement. This is because the property scales with the length scale. This can be illustrated, for example, using the key parameter for heterogeneous catalysts of surface area, *A*, which scales as follows [1]:

(1) $$A\propto {\left(\frac{R}{r}\right)}^{D}r^{2},$$

where *R* is the overall size of the object, *r* is the size of the ruler used to perform the measurement, and the exponent *D* is the (surface) fractal dimension. For a Euclidean, planar geometry, the exponent in Equation (1) would be equal to 2. For a rough surface, the value of *D* falls in the range 2 to 3, where 3 corresponds to a surface so convoluted that it fills the three-dimensional space containing it. Hence, the value of the surface-fractal dimension is a measure of the degree of roughness or convolution of the surface and the extent to which the object fills space. For a flat surface with *D* = 2, the area is ∝*R^2^*, as might be expected. However, if *D* > 2, then the obtained surface area depends upon the size of the ruler (*r*). For ideal, mathematical fractals, the convolutions continue forever down in length scale. However, for real-world objects, there are upper and lower length-scale cut-offs between which the fractal-type scaling law applies. Beyond these cut-offs, the system may behave as if it has a Euclidean geometry, and, for example, the measured surface area does not change with ruler size. The classic real example of this is the length of the coastline of the island of Great Britain, where the obtained value depends upon the length of the ruler used. Using ever shorter rulers would permit the inclusion of smaller and smaller convolutions to the coastline, from major bays, down through coves, to indentations in cliffs, and so on. The obtained length grows as the size of the ruler decreases. In the real world, in the context of catalysts, ‘rulers’ are individual molecular species, such as reactants, transition complexes, and products. A catalyst surface could be potentially tiled with molecular ’tiles’ of size *r^2^*, where this is the cross-sectional area of the molecule (e.g., 0.16 nm^2^ for dinitrogen). A rough surface leads to a form of ‘molecular sieving’ whereby only small molecules can fit in the narrowest convolutions of the surface, while larger molecules are excluded, as shown in Figure 3.

A similar fractal approach can be applied to characterising the space-filling properties of other key parameters of porous heterogeneous catalysts, such as the solid mass or the void space volume [1]. Hence, the use of fractal models is a potential strategy to deal with the high degree of complexity often associated with heterogeneous catalysts, where conventional Euclidean geometry cannot cope. While more traditional catalyst supports, such as sol–gel silicas or precipitated gamma-alumina, are often described as ‘amorphous’ or ‘disordered’, as seen below, these types of materials often actually possess a hidden degree of order or pattern, in the form of the internal symmetry of self-similarity [1]. However, for apparently amorphous materials, this self-similarity is most often of a statistical nature. Statistical self-similarity means that, even though there is (often random) variation in a particular geometry, certain properties averaged over test volumes scale with the length scale of those volumes. Furthermore, fractal models of more amorphous materials include dead-end pores that are typically neglected in more Euclidean models, such as random pore bond networks [1]. 

Even more complex structures, where the fractal dimension exponent in scaling laws like Equation (1) varies depending upon the considered length-scale range, are sometimes called “multi-fractals” [1]. For real catalyst materials, the length-scale at which the change in the scaling-law exponent occurs is generally associated with a characteristic feature of the structure. For example, the scaling-law exponent often changes around the size of the powder crystallites making up an aggregate material because low length-scale fractality is associated with the surface roughness of individual crystallites, whereas larger length-scale fractality is associated with the envelope surface of the pore volume within the packing of the many crystallites forming the aggregate [1]. In a real material with cut-offs, ideally, a fractal scaling law should extend over about an order of magnitude in length scale to be properly valid [1].

## 2. Fractal Theory and Measurement Methods

In this section, only the aspects of experimental techniques relevant to the measurement of fractal dimensions are discussed. For more details on the techniques themselves, the reader is directed to the relevant textbooks and other sources [5,6,7,8].

### 2.1. Gas Sorption

The gas sorption experiment consists of a number of stages characterised by different physical processes that can be used to measure the fractal dimension. At the lowest pressures, the adsorption process consists of individual molecules sticking to the solid substrate itself and eventually filling all the available sites on the surface. Next, a continuous, multi-layer film builds up on the surface. Finally, the core of the pore volume fills with liquid due to capillary condensation.

#### 2.1.1. Molecular Tiling

The basic physical process in gas adsorption is somewhat analogous to the morphological process used to distinguish ideal mathematical objects. Gas adsorption proceeds by progressively increasing the surface coverage of a porous solid (the ‘adsorbent’) with adsorbed gas molecules (the ‘adsorbate’). As mentioned above, the adsorbate molecules act as tiles that can be used to ‘carpet’ the surface in a film that is a monolayer thick. The number of molecules (the ‘capacity’) required to complete the monolayer on a rough surface depends upon the size of the molecule due to the type of molecular sieving effects shown in Figure 3. 

In order to measure the monolayer capacity, it is necessary to employ a model for the adsorption process, such as the Langmuir [5] model for monolayer adsorption or the Brunauer–Emmett–Teller (BET) [9] model of multi-layer adsorption. Langmuir and BET analyses are the most commonly used methods in catalysis to obtain specific surface area from gas adsorption isotherms [8]. These models include the statistical monolayer capacity as one of their parameters and, thus, can be used to measure this quantity. However, they also make several assumptions about the nature of the adsorption process that may not apply to the sample under test. It is assumed that the surface of the adsorbent is homogeneous such that adsorption on any given site is isoenergetic. It is also assumed that the size of the cross-sectional area (the ‘ruler’) does not vary with adsorption capacity. For example, this might not be the case if the conformation of the adsorbate molecule on the surface varies across the surface or with coverage [5,6,7]. Furthermore, specific adsorption may occur such that the apparent monolayer capacity just reflects the high energy adsorption sites on the surface. 

**Figure 4:**
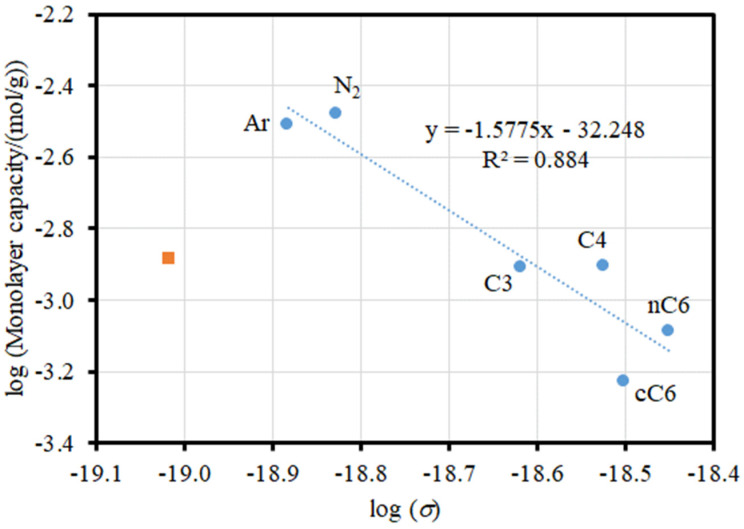
Plot of the logarithm of the monolayer capacities for water (■) and the other (labelled; C3, propane; C4, butane; nC6, n-hexane; cC6, cyclohexane) adsorbates (●) on G1 against the logarithm of the molecular cross-sectional area (*σ* = *r^2^*). The dashed line shows a straight-line fit (with equation and coefficient of determination shown) to the data for the adsorbates other than water. Reprinted with permission from Ref. [10], 2022, Elsevier.

Figure 4 shows an example of the application of Equation (1) to the adsorption of a range of different adsorbates on a sol–gel silica, denoted as G1 [10]. The effective cross-sectional area for each adsorbate was calculated from the molar volume for a liquid under the conditions of the isotherm according to the method described by Gregg and Sing [5]. This presumes that the adsorbed phase is in a liquid-like state, which may not be accurate. In Figure 4, while the data for the other adsorbates broadly follow a linear trend, water is a clear outlier; the monolayer capacity is significantly lower based upon its molar volume and the trend for the other adsorbates. The surface-fractal dimension for G1 derived from the fit of Equation (1) to the data points for the adsorbates other than water is 3.15 ± 0.57, with this unrealistically large value and large error bar for that value (that does include the physically meaningful range of 2 ≤ *D* ≤ 3) resulting from the scatter about the main trend, even for the adsorbates other than water. The scatter may result from the variability in the degree of applicability to each adsorbate of the BET model used to obtain the monolayer capacity. It is likely that the particularly discrepant behaviour of water is due to specific adsorption on patches of surface hydroxyls, which are known to exist on sol–gel silicas [11]. 

#### 2.1.2. Multi-Layer Adsorption

**Figure 5:**
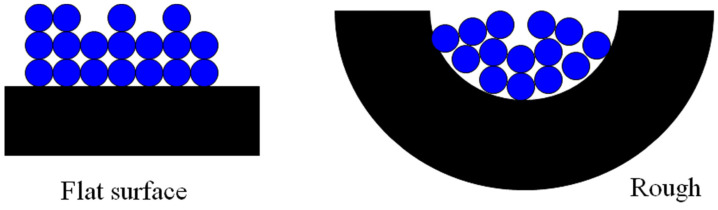
Schematic diagram illustrating the impact of rough surfaces on the surface area available for multi-layer build-up. The number of surface sites stays the same in successive layers for a flat surface, while the number of sites declines with each successive layer for concave surfaces.

In addition to the molecular sieving effect influencing perceived surface area, fractal surfaces also give rise to another effect that impacts the adsorption in the multi-layers above the monolayer. The ‘ruler’ used to measure the surface can be, de facto, the thickness of the adsorbed multi-layer film [1,8]. Due to the (predominant) concavities that exist on the rough surface of a porous solid, the number of available adsorption sites declines in successive multi-layers, as shown schematically in Figure 5. If the surface is fractally rough, then the ratio of the area (*A*) available for adsorption in layer *i* to that in the first layer is given by [1,12]

(2) $$\frac{A_{i}}{A_{1}}=i^{2-D},$$

where *D* is the surface-fractal dimension. 

**Figure 6:**
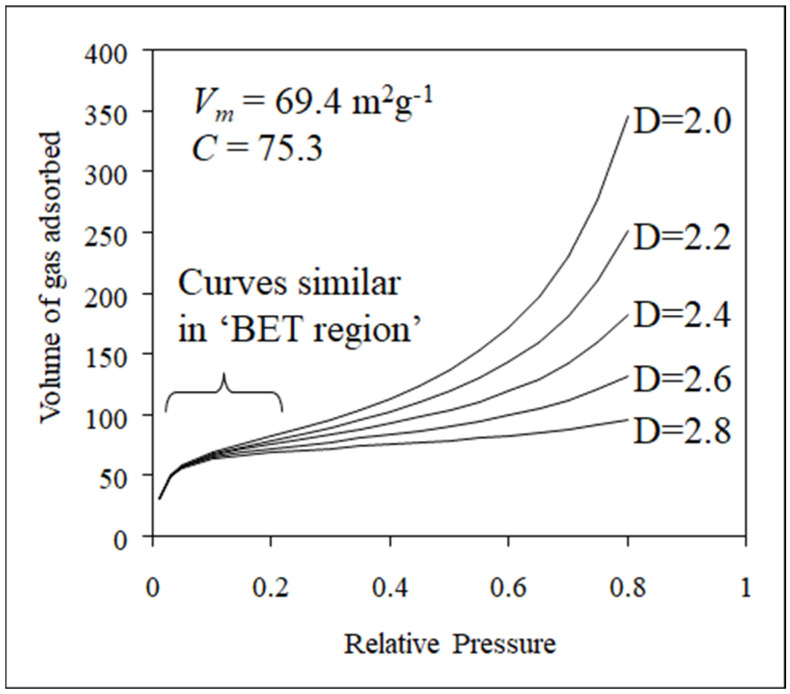
Examples of the isotherms obtained from the fractal BET equation for a range of surface-fractal dimensions. *V_m_* is the monolayer capacity, and *C* is the BET constant. Reprinted with permission from Ref. [8], 2020, Springer-Nature.

The effect given by Equation (2) can be incorporated into the BET model of multi-layer adsorption to obtain a fractal BET equation [12]. Examples of the predicted model isotherms for a variety of surface-fractal dimensions are given in Figure 6. In Figure 6, it can be seen that the isotherms for surfaces with different fractal dimensions are virtually identical in the monolayer region (‘BET region’) but progressively diverge in the multi-layer build-up region as the effect implicit in Equation (2) becomes more apparent for ever higher multi-layers. 

An alternative model for multi-layer build-up on a fractal surface is given by the fractal version of the Frankel–Halsey–Hill (FHH) Equation [13]:

(3) $$ln\left(\frac{V}{V_{m}}\right)=K+Sln\left[ln\left(\frac{P_{0}}{P}\right)\right]$$

where *V_m_* is the monolayer capacity, *K* is a constant, and *P/P*_0_ is the relative pressure. Equation (3) is based upon an adsorption model that treats the adsorbed film as a continuum, so is only strictly valid for adsorption above a monolayer. The *S* parameter is a power-law exponent dependent on *D*, the surface-fractal dimension, and the mechanism of adsorption. There are two limiting cases:  At the lower end of the isotherm, representing the early stages of multi-layer build-up, the film/gas interface is controlled by attractive van der Waals forces between the gas and the solid, which tends to make the film/gas interface replicate the surface roughness. In this case, the value of the constant *S* is given by:

(4) $$S=\frac{D-3}{3}.$$

At higher coverages, however, the interface is controlled by the liquid/gas surface tension, which makes the interface move farther away from the surface so as to reduce the surface area. In this second case, *S* is given by

(5) S=D−3.

In both the cases of the fractal BET and fractal FHH equations, the experimental isotherm data may only obey the fractal model over a limited range of relative pressures. Under both circumstances, when there is a single fractal-scaling regime, the ratio of *V/Vm* is related to the number of adsorbed layers (*n*) as follows [1,13]:

(6) $$n={\left(\frac{V}{V_{m}}\right)}^{1/(3-D)}$$

In conjunction with the size of an adsorbate molecule to give the depth of one adsorbed layer, Equation (6) can be used to calculate the thickness of the adsorbed film for which fractal scaling has been observed (when the data obey the fractal BET and/or fractal FHH equations). For the fit of the model to be physically meaningful, it is suggested that the fractal-scaling region should extend over an order of magnitude in length scale or more [1,8,13]. It is common in the literature for claims of fractal scaling to be only for a limited length-scale range and/or a statistically insignificant level of fit to the fractal law.

As seen below, the surface-fractal dimension obtained from gas adsorption can be compared with that obtained by completely independent physical methods, such as small-angle X-ray scattering (SAXS) (see Section 2.3) [1,11]. It has been found that, for some sol–gel silicas, the surface-fractal dimension obtained by analysis of nitrogen adsorption data using the fractal BET or fractal FHH isotherm models gives rise to values significantly higher than those obtained from both a similar analysis of adsorption data but for butane as the adsorbate and from SAXS [11]. It was suggested that this discrepancy may have arisen because of the specific adsorption of quadrupolar nitrogen on the permanent dipoles of patches of hydroxyl groups on the silica surface obtained by partial dehydroxylation during thermal pre-treatment of the sample prior to adsorption [11]. Patchwise adsorption also gives rise to a (further) decrease in the area available for adsorption in successive adsorbed layers if build-up occurs in pyramidal stacks, thereby leading to an overestimation of the fractal dimension. This work suggests the need to independently validate fractal scaling, as it may only be apparent due to heterogeneity in surface chemistry [11]. 

#### 2.1.3. Capillary Condensation

In addition to the potential ‘rulers’ provided by the size of the molecule itself and the adsorbed film thickness, when the applied pressure in a gas adsorption experiment reaches the level at which capillary condensation starts, this particular physical process furnishes another potential ‘ruler’ in the form of the radius of curvature of the liquid-vapour meniscus in the pores [1,8]. When the pressure of a vapour is raised sufficiently, liquid adsorbate condenses in the whole of a pore. The pressure at which this occurs is given by the Kelvin equation (for an isolated cylindrical pore) [5]:

(7) $$ln\left(\frac{P}{P_{0}}\right)=-\frac{k\gamma \bar{V}}{r_{c}RT}cos\theta$$

where *γ* is the surface tension, $\bar{V}$ is the molar volume, *r_c_* is the radius of curvature of the meniscus (at the boundary of the pore core), *R* is the gas constant, *T* is the absolute temperature, *θ* is the contact angle, and *k* is a constant that depends upon the meniscus geometry. For a meniscus with a cylindrical sleeve geometry, *k* has a value of 1, while for a meniscus of hemispherical geometry, *k* = 2. The Kelvin equation can be used, in conjunction with an isotherm analysis algorithm such as the Barrett–Joyner–Halenda (BJH) algorithm [14], to derive a pore-size distribution in the form of a probability density function for pore size weighted by pore volume. It has also been proposed [1] that for the pore-size distribution of a fractal porous solid, the total volume of pores of diameters ≥ 2r (*V_r_*) obeys the following [1]:

(8) $$-\frac{dV_{r}}{dr}\propto r^{2-D}$$

Therefore, if the pore surface is fractal, a double logarithmic plot of log(*V − V_r_*) against log(*2r*) is linear, with a slope of (*3−D*). This equation is frequently applied to the analysis of pore-size distributions arising from nitrogen sorption capillary condensation region hysteresis loops and mercury porosimetry intrusion curves. Again, this fit needs to be applied across a wide range of length scales (pore sizes in this case) to be physically meaningful and show a statistically significant linear fit. Many datasets in the literature often exhibit a pronounced systematic curvature in the log–log plot, thereby suggesting that a claim of fractal scaling is not really appropriate or is only weakly approximate, at best.

It should be borne in mind that the form of the Kelvin equation given in Equation (7) is for isolated, long, cylindrical pores. The condensation (or evaporation) pressure could well be different if the pores are of differing geometries to that of a cylinder [8]. For irregular pore geometries, the thickness of the adsorbate multi-layer film is not necessarily even throughout, and it can act to fill in surface irregularities at higher coverages, as described by Equation (2). Furthermore, if the surface tension of the adsorbate film is relatively high, then it can also act to smooth the meniscus boundary at the edge of the pore core and make it (approximately) Euclidean, as described by Equation (5).

In principle, the fractal scaling law in Equation (8) could be applied to either or both the adsorption and desorption branches of a gas sorption hysteresis loop. However, either or both of these branches may potentially be affected by pore-to-pore co-operative sorption effects for void spaces consisting of interconnected networks. The adsorption branch may contain advanced condensation and/or network-delayed condensation effects, while the desorption branch may contain pore-blocking and/or cavitation effects [8,15]. These processes, if operating, result in the skewing of the apparent pore-size distribution towards smaller (advanced condensation, pore blocking) or larger (delayed condensation) pore sizes. In such a case, the obtained value of the fractal dimension would be affected by this skew. If it is desired to obtain the surface-fractal dimension from the capillary condensation region of gas sorption data, then it is necessary to utilise the methods developed to test for the presence of these pore-to-pore co-operative effects and alleviate their effects [8,15].

#### 2.1.4. Surface Chemical Heterogeneity

The foregoing models of gas sorption only account for the influence of the surface geometric heterogeneity of fractal catalysts on adsorption and not surface chemical heterogeneity. However, surface chemical heterogeneity over large length scales (much larger than a single adsorption site) can be included using the homotattic (=isoenergetic) patch model [8,16]. This model envisages that the surface consists of a number of different zones, or ‘patches’, within which adsorption is described by a particular isotherm model, which may differ between the various patches. Additionally, it is assumed that the overall size of each of these patches is sufficiently large that any edge effects where they adjoin each other are negligible compared to the bulk behaviour within a patch. If the model isotherm used for each type of zone consists of a fractal BET model, then each patch can have a different surface chemistry characterised via a particular value of the BET constant and a different degree of surface roughness characterised via a particular surface-fractal dimension [17]. Hence, the homotattic patch model can incorporate multi-fractal surfaces of the composite type mentioned in Section 1.

### 2.2. Mercury Porosimetry

As briefly alluded to above, the mercury intrusion curve can be used to obtain the fractal dimension of a porous solid. A variety of methods has been used to relate the mercury intrusion pressure and intruded mercury volume to the void volume, and/or skeletal solid volume, and/or pore surface area of the intruded void space to obtain a fractal dimension. In a mercury porosimetry experiment, fundamentally, PV-type work is done to expand the surface area of the mercury, which is in contact with the interior surface of the porous material. It has been proposed that the variable ‘ruler’ length in mercury porosimetry is, analogous to capillary condensation of vapours, the radius of curvature of the mercury meniscus. The characteristic size of void spaces that mercury can enter is determined by this radius of curvature (*r*), typically given by an equation usually referred to as the Washburn equation [7,8]:

(9) $$r=\frac{-2\gamma cos\theta }{P}$$

where *γ* is the surface tension of mercury, *θ* is the contact angle (usually taken as >90° for a non-wetting fluid—hence, the minus sign), and *P* is the applied hydrostatic pressure in the mercury. Equation (9) is written for straight, uniform cylinders and, thus, implicitly assumes that pore geometry. The fractal dimension *D* can be obtained from the log-log plot of [1,18]:

(10) $$log\left(V_{0}-V_{r}\right)\propto \left(3-D\right)log\;(r)$$

where *V_0_* is the total specific pore volume, *V_r_* is the cumulative intruded volume of pores down to a size of *r*. Depending upon how the intrusion of mercury is conceived [19], the fractal dimension from mercury porosimetry can be a surface and/or pore-fractal dimension. In many cases, the surface-fractal dimension of a pore fractal is the same as the pore-fractal dimension. Mercury intrusion porosimetry can only be used to obtain accurate fractal dimensions for certain types of structures, such as a fractal ‘tree-type’ structures, for reasons that will be explained below.

A key issue with using mercury porosimetry to obtain fractal dimensions is that the intrusion curve can be (incorrectly) skewed to smaller pore sizes due to the pore-shielding (or pore-shadowing) effect [7,8,20]. This arises in situations analogous to the so-called ‘ink-bottle’ pore geometry, where a large pore body can only be accessed from the exterior via a much narrower pore neck. This means that in mercury intrusion, the pressure must be increased to allow mercury to enter the neck before it can also enter the body at the same time. Hence, the volume of the pore body can be attributed to pores of the size of the neck. This effect leads to the PSD being skewed towards smaller pore sizes in a similar way to the pore-blocking effect in gas desorption.

However, there are ways to check for the presence of pore-shielding effects. The influence of contact-angle hysteresis effects can be removed through the use of an independently calibrated alternative to the Washburn equation, such as those proposed by Kloubek [21] for silicas and Rigby [8] for aluminas. If, after using the calibrated equations, there is still residual hysteresis, this is likely to be structural and, thus, indicative of pore-blocking effects. For example, it was found that, using the Kloubek [21] correlations, the mercury intrusion and extrusion curves for powdered samples of sol–gel silica spheres could be completely superposed, indicating that the contact-angle hysteresis had been completely removed and no residual structural hysteresis remained. Hence, the true pore-size distribution could be obtained.

### 2.3. Small-Angle X-Ray Scattering (SAXS)

The intensity of X-ray radiation scattered by a fractal surface is known to be proportional to a negative power of the *q*-wave vector such that [1,8,22,23]

(11) $$I\left(q\right)=q^{-\eta },$$

where *q* = 4*πλ*^−1^ *sin*(*φ*/2), *λ* is the wavelength of the radiation, and *φ* is the scattering angle. Normally, this behaviour is only observed if *q* satisfies the inequality of *qξ* >> 1, where *ξ* is the characteristic length scale for the structure creating the scattering. From the value of *η*, the fractal nature of the system under investigation can be determined [1,23]. If the exponent is in the range of 1 < *η* < 3, then it describes the mass fractal of dimension (*D_m_*)SAXS = *η*, but if in the range is 3 < *η* < 4, then it describes surface fractals of dimension (*D_S_*)SAXS = 6 − η. For *η* = 4, Equation (11) leads to Porod’s law, where (*D_S_*)SAXS = 2 and the surface is flat.

A key difference between adsorption and scattering methods is that the former only provides the fractal characterisation for the surface area accessible from the exterior, while the latter can also measure the surface-fractal dimension for closed (disconnected) void spaces. This difference in sampling can be advantageous and exploited, such as when surfaces accessible from the exterior have been modified.

### 2.4. NMR

The relaxation rate of NMR active nuclei within solids due to the presence of paramagnetic impurities is affected by the relative juxtaposition of such centres in space around the relaxing nucleus. If the solid, such as a porous glass, has a fractal mass distribution and the paramagnetic centres have a uniform distribution within the solid, then the NMR active nuclei and paramagnetic centres will have a fractal distribution in space, which affects the variation of concentration of particular sites with the distance from other sites, which, in turn, can affect the observed relaxation rate of NMR active nuclei [24].

Sapoval et al. [25] showed that the NMR surface relaxation rate of fluids within void spaces can be affected by the irregular geometry of fractal pores depending upon the relative rates of relaxation and diffusion to the surface. For relatively fast relaxation, the observed rate depends upon the fractal dimensionality, whereas for slow relaxation, relative to diffusion, the fractality has little impact.

### 2.5. Microscopy and Imaging

#### 2.5.1. Imaging Modalities

**Figure 7:**
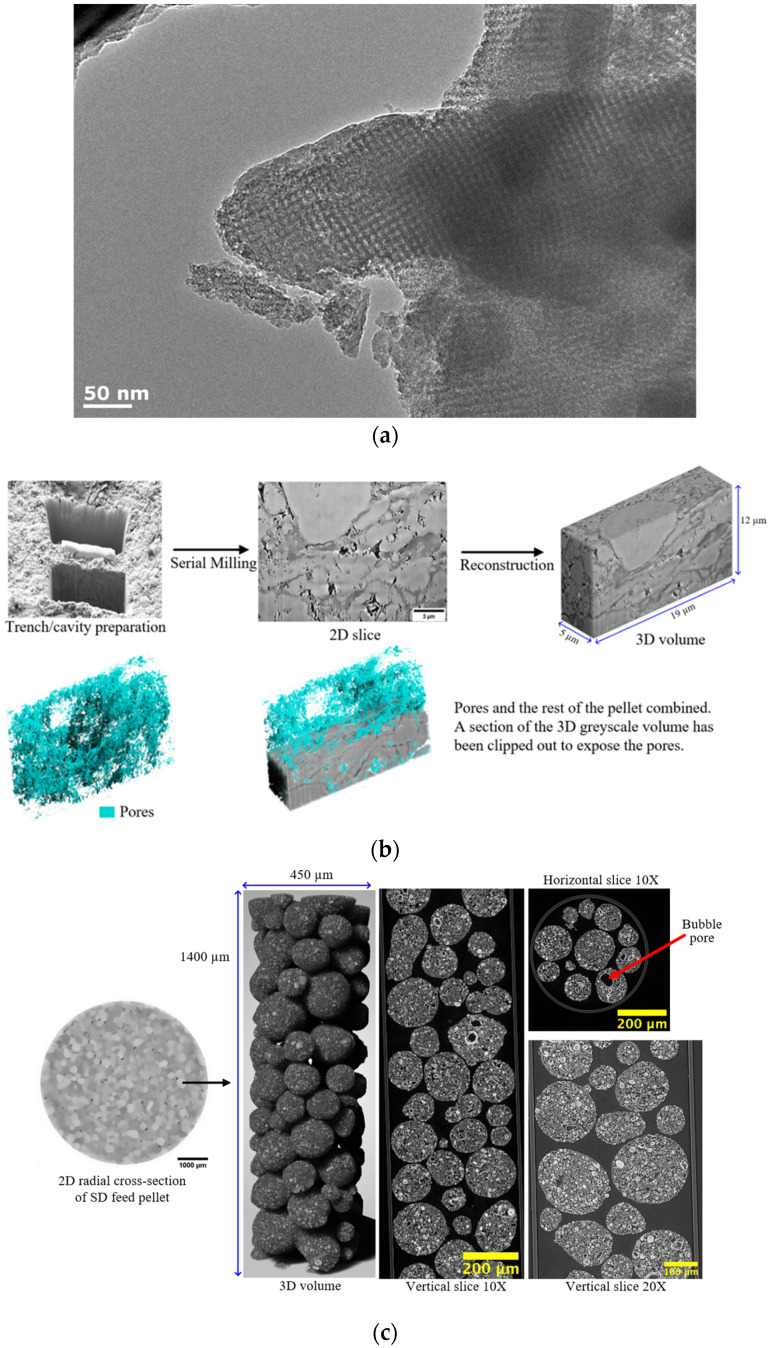

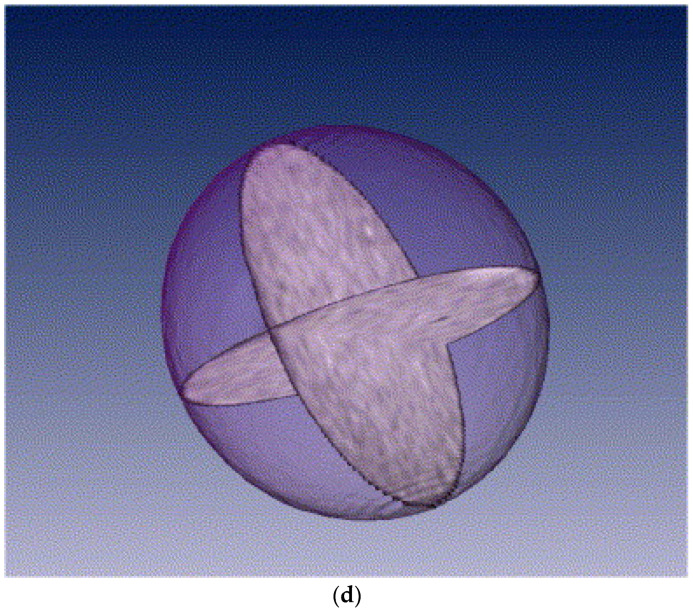
(**a**) TEM image of SBA-16 silica material. The square array of white dots in the centre of the image corresponds to pore bodies formed by the (subsequently removed) polymer template. Reprinted with permission from Ref. [8], 2020, Springer-Nature. (**b**) 2D and 3D reconstructed greyscale FIB-SEM images and segmentation result for fresh spray-dried methanol synthesis catalyst pellet. Also shown in the figure is the trench/cavity site. The scale bar corresponds to 3 μm. A denser spheroidal region is evident from the void amidst the scatter of (blue) macropores picked out by image segmentation. (**c**) 2D radial cross-sections and 3D reconstruction of high-resolution CXT image of spray-dried (SD) feed particle used to make the SD feed catalyst pellet. Also shown on the left side of the figure, for comparison purposes, is a low-resolution image of a whole SD feed pellet, with an arrow indicating a corresponding individual constituent feed particle. The fractal-like nature of the structure is evidenced by the visual similarities of the low- and high-resolution images. Reprinted with permission from Ref. [27], 2023, Elsevier. (**d**) MRI spin–spin relaxation time images of perpendicular 2D slices through the centre of a sol–gel silica catalyst support pellet. The pixel resolution is 40 μm, and the slice thickness is 250 μm. Reprinted with permission from Ref. [28], 2006, Elsevier.

A variety of imaging modalities have been applied to examine catalyst materials [8]. These include transmission electron microscopy (TEM) and its extension, electron tomography (or 3D-TEM); scanning electron microscopy (SEM) and its extension, focused ion beam (FIB)-SEM; computerised X-ray tomography (CXT) (or CT-scanning, microfocus X-ray (MFX) imaging); and magnetic resonance imaging (MRI). These various imaging modalities can produce several different types of datasets, as shown in Figure 7. For example, depending on whether the pores in the sample material are filled with a contrast agent (e.g., a resin) and serial-sectioned before imaging or the surface is roughly fractured, SEM can produce density-dependent greyscale images of well-defined planes through the sample using back-scattered electrons, or a particular perspective view of the surface topography of the material using secondary electron imaging. CXT and MRI can produce full-volume imaging of void spaces where the individual pores are larger than the imaging resolution. SEM, MRI, and CXT of materials with pores below voxel resolution can still produce full 2D or 3D lattice-based images where the individual pixel/voxel intensity is weighted in some way by the underlying voidage fraction within the voxel volume [26,27,28] or, for MRI, the pore size or connectivity (via relaxation time (see Figure 7d) or diffusometry-weighted imaging, respectively). The choice of particular fractal-based image analysis method depends upon the nature of the image. Given that fractal scaling should extend over a wide range of length scales, multiple imaging modalities appropriate to different length scales can be used to study the same porous catalyst sample or material.

#### 2.5.2. Image Analysis

Fractal-based image analysis can involve the measurement of boundaries identified (somehow) within the image or of the spatial distribution of pixel/voxel intensities. Fractal-based image analysis methods have been extensively reviewed elsewhere [29,30], so only a brief summary is provided here. In order to use fractal-based image analysis algorithms on an image, some pre-processing is often also required. It may be necessary to remove artifacts or noise in the image using suitable filters. In addition, for direct imaging of pores or other defined features (e.g., metal crystallites) in images, in order to apply analysis algorithms that only work with binary images, it is also necessary to segment (or gate) the image into two different phases. These processes have their own associated issues, which have also been reviewed elsewhere [30,31]. In particular, the segmentation of an image can be particularly problematic for fractal-based image analysis algorithms. 

The boundary to be characterised can be identified by the segmentation procedure; for greyscale images, alternative methods exist. This method involves treating the greyscale as if it were the relief of a surface and finding the contour lines (isarithms) of the greyscale spatial distribution [29,30]. For example, Baldwin et al. [32] used a boundary extraction, or isarithm method where ever-increasing bins in image intensity were used, with a fixed upper bound above the maximum in image intensity, and variable lower bound. Clusters of nearest neighbouring pixels with intensities within the current bin were identified, and the areas and perimeters were measured. The perimeters of the clusters formed the successive isarithms or contours of the grey-level surface. Since the contours are drawn around regions of images containing pixels with intensity values above a particular cut-off, noise in the pixel values only has a marked effect if the bin size of the gaps between contours is too small relative to the noise. This can be tested by varying the bin size and checking for any effect on the measured fractal dimension. The fractal parameters derived from MR images (like that in Figure 7d) in this way can be correlated with the transport properties of catalyst pellets [33].

Many fractal image analysis algorithms are based upon the effect of a variable ruler size, as mentioned above. Structured walk methods for the measurement of the fractal dimension of boundaries are based upon a variable ruler size. This involves mimicking the walking of dividers along a boundary using a computer simulation. However, a major issue associated with implementing this method on a computer is the necessity for the digitization of the boundary such that it is represented by a set of points. If the boundary is irregular, then there may not be a digitization point exactly one (divider/ruler) step away from the current point. 

Accurate digitization of boundaries is crucial for reliable fractal dimension measurements, and the digitization process can introduce errors, particularly when dealing with complex or highly irregular boundaries typical of fractal structures [29].

Potential biases introduced during boundary digitization include the following:

- Pixelation effects (quantization errors);
- Resolution limitations;
- Noise and artifacts;
- Thresholding errors;
- Sampling bias.

Interpolation methods can mitigate these biases without distorting measurements of fractal parameters. Interpolation methods enhance the representation of boundaries by estimating values at sub-pixel levels, effectively increasing the apparent resolution and smoothing out artifacts without distorting the fractal measurements [34,35,36]. They help in the following ways:

1. Sub-Pixel Edge Detection:
  - Improved Edge Localization: By analysing the intensity gradients in the image, sub-pixel edge detection methods can estimate the positions of edges with greater precision than the pixel grid allows.
  - Reduced Quantization Error: This leads to a more accurate representation of the boundary, mitigating the “staircase” effect without altering the fundamental structure.
2. Anti-Aliasing Techniques:
  - Smoothing Stair-Step Artifacts: Anti-aliasing methods adjust pixel intensities to smooth out the abrupt changes along digitized edges, providing a closer approximation of the true boundary.
3. Edge-Smoothing Filters:
  - Noise Reduction: Filters like Gaussian or median filters can reduce random noise in the image, preventing it from affecting boundary extraction.
  - Preservation of Structural Features: Properly designed filters can smooth the boundary while preserving important structural details necessary for accurate fractal analysis.
4. Contour Interpolation:
  - Reconstructing Continuous Boundaries: Interpolation can be used to reconstruct the boundary as a continuous curve, which can then be analysed using methods suitable for continuous data.
5. Adaptive Thresholding:
  - Local Threshold Adjustments: Instead of using a global threshold, adaptive thresholding considers local variations in intensity, improving the accuracy of boundary detection in images with uneven illumination or contrast.

To avoid distorting fractal measurements, it is essential to ensure that interpolation methods do not significantly alter the geometric features contributing to the fractal nature of the boundary. This involves the following:

- Preserving scaling properties;
- Avoiding over-smoothing;
- Cross-validating with original data.

A key further variable ruler-based example is the so-called box-counting algorithm. In this approach, the embedding 2D or 3D lattice containing a boundary is ‘paved’ with a lattice of boxes of successively larger side length (*l* = 1/*δ*). For each box side length, the number (*N*) of boxes containing a part of the boundary being characterised is counted. The fractal dimension (*D*) is then obtained from the following expression [1,29]:

(12) $$D=\underset{\delta \to 0}{\lim}log\left[N\left(\delta \right)\right]/log\left[\delta \right]$$

While the box-counting method traditionally assumes self-similarity, it can be adapted to handle real-world materials that exhibit self-affinity or multifractal characteristics across different length scales. In materials with self-affine properties, scaling behaviour differs along different axes, and this anisotropy can be captured by modifying the box-counting method to use boxes with different scaling ratios along each axis. By analysing the scaling exponents in each direction, one can determine the self-affine fractal dimensions. The box-counting method can identify materials exhibiting multifractality, where the fractal dimension varies with location or scale, by plotting log *N*(*δ*) versus log(*δ*) and observing changes in the slope across different scales. This indicates multiple scaling regimes, each characterised by its own fractal dimension. By calculating the generalized fractal dimensions (*D_q_*) using the multifractal box-counting method, one can obtain a spectrum of dimensions that describe the distribution of scaling behaviours in the material [37]. These adaptations allow the box-counting method to be effectively applied to complex, real-world materials, providing valuable insights into their structural characteristics.

Some fractal-based image analysis algorithms do not involve varying the ruler sizes applied to the image features but, instead, varying the overall size of the objects extracted via image analysis. The aforementioned boundary extraction method proposed by Baldwin et al. [32] described above identifies clusters of increasing size by progressively lowering the lower cut-off in the bin of image pixel intensities. The algorithm then takes advantage of the fact that the area (*A*) and perimeter (*C*) of fractal objects with irregular outlines scale with size via power laws involving the fractal dimension such that

(13) $$A\propto C^{2/D}$$

The fractal dimension can be obtained from a log *A* versus log *C* plot for the pairs of values from all of the clusters identified in the image analysis, except for the smallest clusters, which tend to only exhibit Euclidean-type behaviour (*D*→1). 

The so-called blanket algorithm is another matrix-based method according to which the boundary profile is sequentially coated (blanketed) with successive layers of pixels [1,29,30]. The boundary length can then be estimated at each swelling stage by dividing the total new area of the swollen boundary by the new thickness of the swollen boundary.

A further type of image analysis algorithm that can be applied to greyscale images is fractional Brownian motion-based methods [30]. The variogram method is based upon the statistical Gaussian modelling of images. However, it uses the reverse of the normal forward method that uses a fractal dimension to create an artificial image using fractional Brownian motion to obtain the fractal dimension that would have led to the analysed image. In contrast, the power spectrum method involves Fourier transforming each image line, and the power spectrum thereby obtained is evaluated, and all the power spectra for the whole image are averaged. The fractal dimension is computed from these data [30].

## 3. Synthesis and Fabrication of Fractal Catalytic Materials

Fractal structures in catalytic materials can form either naturally, during specific synthesis processes, or be intentionally engineered using targeted strategies. Understanding the conditions and methods that lead to fractal formation is crucial in the design of catalysts with enhanced performance. In this section, we discuss various synthesis methods that result in fractal catalytic materials, providing detailed explanations and examples from the literature.

### 3.1. Controlled Aggregation and Growth Processes

Fractal structures often emerge naturally during controlled aggregation and growth processes, such as diffusion-limited aggregation (DLA), and cluster–cluster aggregation during sol–gel processes (see Figure 2), where particles aggregate in a manner governed by diffusion rates and reaction kinetics [1]. These aggregation processes can readily be simulated on computers to assess the impact of various different parameters characterizing the process; then, these parameters can be adjusted in ‘real-world’ production settings [1]. For example, DLA starts with a seed particle located in the centre of a lattice of the required geometry (e.g., 3D cubic). Then, additional particles are introduced to the lattice at a given distance from the incipient cluster. They are allowed to perform a random walk on the lattice until they encounter the cluster, whereupon they can stick to it (and become part thereof) with a given probability or continue the random walk until they encounter the cluster again and stick to it that time. In such a manner, the cluster grows. The sticking probability in the ‘real world’ can be varied by changing the surface forces between particles, such as by changing the pH of the solution in liquid systems. The nature of the random walk can be changed by altering the surrounding fluid density and viscosity. Besides aggregation, diffusion-limited deposition (DLD) processes can also be simulated, except that the random walkers first stick to one side of the lattice (representing the bottom of the container) [1]. A bias or drift can be introduced into the random walk to account for gravity or surrounding fluid flow, if necessary. DLD necessarily produces an anisotropic structure, and at least two scaling exponents (fractal dimensionalities) are needed to describe the self-affine structure of the deposit. DLD is frequently used to model the initial stages of production of precipitated alumina supports [1]. 

Coppens [38] employed sol–gel synthesis to fabricate fractal porous catalysts, targeting amorphous materials such as PtRe/Al_2_O_3_, which are essential for industrial applications like the catalytic reforming of naphtha. In the sol–gel process, hydrolysis and condensation reactions of metal alkoxides lead to the formation of a three-dimensional network. The randomness of these reactions results in the formation of hierarchical pore structures with complex, fractal internal surfaces, thereby facilitating enhanced catalytic performance. The synthesized materials exhibited fractal dimensions ranging from 2.0 to 2.67 across length scales of approximately 3 Å to 7 nm, as determined by small-angle and wide-angle X-ray scattering (SAXS and WAXS, respectively). Coppens observed that scattering techniques provided a direct measurement of fractal geometry, thereby outperforming adsorption-based methods such as the BET equation. Adsorption methods can overestimate fractal dimensions due to potential surface dehydroxylation (and, thus, higher chemical heterogeneity) following higher thermal pre-treatment temperatures [11]. In comparing fractal dimension determination methods, Coppens highlighted the superiority of SAXS and WAXS over adsorption techniques for their materials, noting that the latter are influenced by adsorption phenomena rather than purely geometric surface roughness.

While Coppens [38] demonstrated the use of techniques like SAXS and WAXS in determining fractal dimensions, such methods have limitations in fully capturing the complexity of hierarchical or composite porous structures. Imaging modalities often struggle with field-of-view constraints and resolution limitations, which become particularly problematic when characterizing materials with multiscale porosity, as seen in many industrial catalysts [8,27,39,40,41]. In contrast, indirect methods such as gas overcondensation and mercury porosimetry provide a more statistically representative characterisation of the pore structure. These techniques span a wide range of pore sizes, from nanopores to macropores, and are less affected by the spatial limitations of imaging, offering a more exhaustive analysis of the void space and its impact on mass transport properties. Thus, a more comprehensive understanding of pore structure–transport relationships is often yielded, especially for disordered, heterogeneous materials.

Building upon Coppens’ work, Waite et al. [42] investigated the aggregation kinetics and fractal structures of γ-alumina assemblages under varying electrolyte conditions to understand the influence of surface forces on aggregate formation. Fractal aggregates were synthesized by inducing the aggregation of γ-alumina particles in the presence of different anions, specifically chloride, nitrate, and sulphate ions. Notably, very high concentrations of chloride and nitrate anions (>0.5 M) were required to initiate rapid aggregation, which is attributed to strong repulsive forces arising from highly charged Al_13_ polymeric species adsorbed on the γ-alumina surface at slightly acidic pH levels. In contrast, the introduction of sulphate ions at much lower concentrations (1–2 mM) negated these repulsive forces, leading to rapid aggregation. To determine the fractal dimensions of the resulting aggregates, static light-scattering techniques were employed, analysing the power-law behaviour of scattered light intensity as a function of the scattering wave vector. The obtained fractal dimensions ranged from 1.85 to 2.25. Interestingly, unlike typical colloidal systems destabilized by indifferent electrolytes, where lower fractal dimensions are associated with rapid (diffusion-limited) aggregation and higher dimensions with slower (reaction-limited) aggregation, the results did not conform to this trend. Instead, relatively constant fractal dimensions (2.10 to 2.25) were observed over the range of salt concentrations where the transition from slow to rapid aggregation occurred, with a slight increase in the fractal dimension corresponding to higher aggregation rates. Aggregates formed in the presence of sulphate exhibited significantly lower fractal dimensions (1.85 to 1.91) compared to those formed with chloride or nitrate ions.

Following these insights, Rožić et al. [43] explored the surface-fractal dimensions obtained from gas adsorption of activated alumina catalyst supports. It was found that the surface-fractal dimension increased with the temperature of thermal pre-treatment. This finding seems counterintuitive, since, typically, the surface of thermally sintered systems might be expected to become smoother as heated, thereby reducing surface energy. This apparent anomaly may have occurred because the hyperbolic form of the top of the adsorption isotherms and narrow hysteresis in this region suggest that some of the macropores were left unfilled by capillary condensate at the top of the isotherm. The use of the overcondensation technique [8] would reveal these missing pores and enable a more accurate pore-size distribution (PSD) to be determined. In addition, the nitrogen fractal dimensions obtained from the fractal BET equation were higher than those obtained via the pore-size distribution (from the capillary condensation region). This may have arisen because, as the surface is thermally treated to higher temperatures, it can partially dehydroxylate more. If, in the multi-layer region of the isotherm, the nitrogen tends to specifically adsorb in pyramidal stacks on shrinking patches of remaining hydroxyls, it may seem like the fractal dimension is increasing, even though the surface roughness is not actually changing [11].

### 3.2. Template-Assisted Synthesis Methods

Fractal structures can be intentionally engineered by using templates with fractal geometries. This method allows for precise control over the fractal dimensions of the resulting materials. Expanding on the theme of controlling fractal dimensions, Mayama and Tsujii [44] developed a novel template method to synthesize Menger sponge-like fractal silica bodies with controllable fractal dimensions using alkylketene dimer (AKD) particles, which spontaneously formed fractal surfaces. “Fractal AKD particles” were prepared as templates, densely packed, and compressed under varying ratios (1.0, 2.0, and 3.0). The voids between the compressed particles were filled with a tetramethyl orthosilicate (TMOS) solution. After solidification via a sol–gel process and subsequent calcination to remove the AKD templates, silica bodies with cross-sectional fractal dimensions (*D_cs_*) ranging from 1.87 to 1.80 were obtained. These dimensions directly correlated with the compression ratios, demonstrating precise control over fractality.

However, the study’s assumption of self-similarity in the generated fractal bodies may limit the generalizability and applicability of the findings, as real-world catalytic surfaces often exhibit self-affine and multifractal geometries due to anisotropic growth conditions and hierarchical organization at multiple scales. To more accurately reflect these complexities, future template-based synthesis methods could integrate self-affine and multifractal geometries.

One approach is to design templates with anisotropic features or to employ dynamic assembly processes under non-equilibrium conditions to induce anisotropy and heterogeneity in the resulting structures [1,45]. Another strategy involves utilising natural templates that possess inherent multifractal properties, such as the intricate networks found in leaf skeletons or other biological structures. These templates can transfer their complex geometric patterns to the synthesized materials, resulting in highly sophisticated architectures [46]. Additionally, advanced fabrication techniques like additive manufacturing or layer-by-layer assembly allow for precise control over the material structure at different length scales, enabling the creation of self-affine and multifractal architectures [47]. Post-synthesis treatments, such as directional etching or surface modification, can further introduce anisotropy and modify the fractal characteristics of the material. Computational modelling and simulation can also play a crucial role in guiding the synthesis process by predicting the conditions necessary to achieve desired fractal geometries [1]. By moving beyond the assumption of self-similarity, these strategies enable the development of catalysts that more closely mimic the complexity of natural systems, potentially leading to enhanced catalytic performance due to improvements in mass transport properties and active site accessibility.

Subsequently, Yamaguchi et al. [48] further advanced the understanding of fractal porous silica materials by investigating self-assembled structures synthesized via a sol–gel method using AKD as a template. The AKD molecules formed fractal structures through self-assembly, which were replicated in the silica matrix upon calcination. This process resulted in porous silica with mass-fractal structures spanning length scales from approximately 100 nm to 10 μm. The hierarchical structure comprised both mass-fractal and surface-fractal characteristics, with primary silica particles smaller than 10 nm forming a continuous silica matrix. To characterise these complex structures over such a wide range of length scales, a combined small-angle scattering (CSAS) method was employed, integrating ultra-small-angle neutron scattering (USANS), small-angle neutron scattering (SANS), and small-angle X-ray scattering (SAXS). Consistent mass-fractal dimensions of approximately 2.67 and 2.29 were determined for samples with silica volume fractions of 0.15 and 0.04, respectively, indicating differences in structural compactness due to differences in the compression ratios of the AKD moulds.

Building upon the template method introduced by Mayama and Tsujii [44], Ono et al. [49] characterised the structural properties of fractal porous silica synthesized using this approach. Using AKD particles that spontaneously form fractal surfaces as templates, Menger sponge-like fractal silica bodies were created. By stacking these fractal AKD particles with varying compression ratios (1.0, 2.0, and 3.0) and performing sol–gel synthesis with TMOS, silica structures with hierarchical pore sizes ranging from approximately 1 nm to 100 μm were produced. The fractal dimensions of these structures were determined using multiple techniques, including mercury porosimetry, ^1^H NMR cryoporometry, nitrogen gas adsorption, and direct evaluation via the box-counting method applied to SEM images. Interestingly, the cross-sectional fractal dimensions (*D_cs_*) did not show a clear dependence on the compression ratio, contrasting with previous findings where fractal dimensions decreased with increasing compression. Instead, the porosity increased with higher compression ratios, suggesting that compression primarily affected porosity rather than fractality. This discrepancy may have resulted from experimental errors or anisotropy in the template particles upon compression. Furthermore, it was observed that the pore volume distribution and specific surface area could be discussed in terms of different fractal porous structures at distinct scale regions. Two porous structures were identified: a Menger sponge-like structure in the range of approximately 100 nm to 10 μm and intrinsic pores around 4 nm formed during the sol–gel process. The larger fractal pores contributed significantly to the pore volume but not to the specific surface area, which was mainly determined by the smaller intrinsic pores. The calcination temperature also influenced the porous structure, particularly affecting the smaller pores below 10 nm due to shrinkage and collapse of the silica network at higher temperatures.

Shifting focus back to bioinspired synthesis methods, Sharma et al. [46] developed a method for fabricating high-surface-area CuO microcactus structures on Bauhinia racemosa leaf skeletons, creating efficient biotic dip catalysts. A combination of sputtering, electrodeposition, and chemical oxidation techniques [50] was employed to uniformly coat the leaf skeletons with CuO, resulting in a fractal-like hierarchical morphology resembling the Mammillaria prolifera cactus. The unique multiscale surface features of the leaf skeletons played a crucial role in determining the morphology of the final CuO microstructure. Characterisation techniques including SEM, EDS, XRD, and XPS confirmed the presence of crystalline monoclinic CuO with a high degree of purity. The CuO microcactus-bearing leaf skeletons exhibited a high surface area of 3.09 ± 0.03 m^2^ g^−1^, as determined by BET analysis. This bioinspired synthesis route offers a sustainable and scalable method for designing hierarchical catalysts with improved performance, highlighting the potential of natural templates in the development of fractal catalytic materials. However, further quantitative analysis of the fractal characteristics would be beneficial for a more comprehensive understanding and comparison with other fractal-based systems.

### 3.3. Electrochemical Deposition Techniques

Fractal dendritic structures can form during electrochemical deposition under specific conditions, such as high overpotentials or low electrolyte concentrations. By manipulating parameters like current density, electrolyte concentration, and additives, fractal morphologies can be engineered to enhance catalytic performance. Advancing the synthesis of fractal materials, Cheng et al. [51] reported a facile one-pot method for the synthesis of Pt nanodendrites with highly open 3D structures, which exhibited enhanced electrocatalytic activity and stability towards the methanol oxidation reaction (MOR) and oxygen reduction reaction (ORR) compared to commercial Pt/C catalysts. By utilising K_2_PtCl_4_ as the metal precursor, L-ascorbic acid as the reducing agent, and hexadecyltrimethylammonium chloride as the capping agent in an aqueous solution at 96 °C for 30 min, they prepared Pt nanodendrites characterised by branching structures at multiple length scales—a fractal-like morphology leading to a high surface area and an increased number of active sites.

The fractal geometry of these nanodendrites plays a crucial role in enhancing their electrocatalytic properties by creating a hierarchical structure with abundant accessible active sites and efficient pathways for mass transport. This fractal-like morphology ensures that active sites are available at different scales, improving catalyst utilisation and facilitating rapid diffusion of reactants and products. The open 3D structure provides interconnected pathways for electron transfer and reactant diffusion, further enhancing catalytic efficiency. Cheng et al. [51] reported that the electrochemically active surface area (ECSA) of Pt nanodendrites was 117 m^2^ g^−1^Pt, which is significantly higher than the 83 m^2^ g^−1^Pt for commercial Pt/C catalysts, highlighting the role of fractal geometry in increasing the availability of active sites. Compared to non-fractal nanostructures, the fractal nanodendrites demonstrated superior performance due to their unique structural features that improve catalytic activity and stability

### 3.4. Self-Assembly Processes

Fractal structures can emerge naturally from self-assembly processes driven by specific interactions among nanoparticles, influenced by parameters such as ionic strength, pH, and the presence of surfactants. Wang et al. [52] synthesized porous alumina using carbon fibres and graphite as pore formers. Mercury intrusion porosimetry (MIP) analysis revealed bimodal or trimodal pore size distributions (PSDs) at 50 vol.% of pore formers and unimodal PSDs at 30 vol.% and 70 vol.%. Surface-fractal dimensions (*D_s_*) were determined from MIP data using the Zhang–Li model [53], showing *D_s_* values between 2.4 and 2.7. *D_s_* decreased with increasing pore-former content, correlating with physical properties. The self-assembly of particles led to fractal structures with enhanced surface areas, contributing to improved catalytic performance.

### 3.5. Controlled Precipitation and Crystallization

As mentioned briefly above, fractal structures can emerge during precipitation or crystallization processes when supersaturation levels, temperature, and additives are carefully controlled, influencing the nucleation and growth of crystals. More recently, Ying et al. [54] introduced a method for synthesizing hierarchical fractal PtPd nanoparticles using pyridinium-type ionic liquids as structure-directing agents. This approach controlled particle and pore sizes through self-similar growth, producing materials with pores between 1.4 and 1.8 nm. Characterisation techniques such as HAADF-STEM, TEM, and XRD confirmed alloyed nanostructures with highly exposed active sites. Although fractal dimensions were not explicitly measured, molecular dynamics simulations provided insights into their formation. Notably, these materials exhibited exceptional hydrothermal stability, maintaining integrity after treatment at 100 °C for 96 h, contrasting with other methods under which fractal stability declines under similar conditions.

### 3.6. Fractal Structures Emerging from Fabrication Methods

Fractal structures can also emerge naturally from fabrication methods involving recursive processes, such as tableting with feeds formed by spray drying or roll compaction, leading to hierarchical pore structures spanning multiple length scales. Mousa et al. [27,41] investigated the synthesis, characterisation, and evolution under reaction conditions of methanol synthesis catalyst pellets formed by the compaction of feed particles fabricated via spray drying (SD) or roll compaction (RC). By using multimodal imaging techniques, including high-resolution computerised X-ray tomography (CXT) and focused ion beam-scanning electron microscopy (FIB-SEM), together with multiscale porosimetry methods such as gas overcondensation and mercury porosimetry, it was revealed that both SD and RC pellets exhibited fractal-like, hierarchical pore structures spanning from nanometres to millimeters. For example, the nearly spherical SD feed particles making up the whole pellet seen in low-resolution CXT images are, themselves, composites made up of smaller, roughly spherical particles, as seen in high-resolution CXT images (see Figure 7c). A pre-compaction step in the fabrication of RC feed particles also led to a fractal-like structure for the finished pellet, since the internal structure of the individual RC feed particles resembles that of the internal structure of the finished pellet, since both are formed by smaller, irregular particles.

In an initial study [27], fresh SD and RC pellets were characterised, and it was found that the fractal pore networks resulting from the fabrication method significantly influenced mass transport properties. This is an example of how fractal-like recursive fabrication methods give rise to fractal pore networks [1]. In a subsequent study [41], the evolution of these pore structures during catalyst reduction and sintering was examined, focusing on reduced and aged (spent) catalysts. It was discovered that reduction and sintering induced new macroporosity due to solid shrinkage and reorganization, which disproportionately affected mass transport compared to the previous pore volume fraction. By applying percolation theory and random pore-bond network models, it was demonstrated that these induced macropores become the critical pathways for mass transport, thereby altering the identity of the most important pore structural features, especially in RC pellets. Together, these studies emphasize the importance of fabrication methods and processing conditions for the hierarchical pore structures and mass transport behaviour of catalyst pellets. These studies, like the MR image in Figure 7d, show the importance of macroscopic (>10 μm) structural heterogeneity to mass transport in formed catalyst materials and how this heterogeneity can be fractal in character or characterised via fractal concepts [1,27,33,41].

### 3.7. Fractal Characteristics in Zeolites

Given the limited, fixed-length scale typical of the unit cell for crystalline zeolites, it might be expected that zeolites do not possess significant surface-fractal dimensionality. Indeed, this is what has been found for zeolites by some workers [38]. However, the molecular sieving properties of zeolites might lead to the sensitivity of the measured characteristics to the size of the used molecular probe. In modelling work, Torrens [55] found that, while most zeolite cavities do not show any fractal character, zeolites with six to eight ring cavities did have fractal dimensions above the lower Euclidean bound. Furthermore, one might anticipate that, if zeolites are subjected to treatments that increase the degree of disorder, like de-alumination, then some surface roughness and void space heterogeneity would be introduced that might result in fractal scaling.

In summary, fractal structures in catalytic materials can arise both naturally during specific synthesis procedures and through intentional engineering. Understanding the synthesis strategies and conditions that lead to fractal formations allows for the design of catalysts with tailored properties and enhanced performance. The discussed methods—ranging from controlled aggregation and template-assisted synthesis to electrochemical deposition and self-assembly—provide valuable insights into the formation of fractal catalytic materials. By leveraging these strategies, researchers can develop catalysts with improved mass transport properties, greater active site accessibility, and enhanced catalytic activity, addressing key challenges in heterogeneous catalysis.

## 4. Use of Fractal Materials in Heterogeneous Catalysis

A series of molybdenum nitride catalysts were synthesized by nitriding a mixture of molybdenum oxide and alumina at a range of different temperatures [56]. The surface-fractal dimensions of these materials were determined using the molecular tiling method involving nitrogen and a series of short-chain alkanes. It was found that the surface-fractal dimension generally increased with the catalyst fabrication nitridation temperature until 1073 K; then, it declined. It was also found that the subsequent hydrogenation activity of the catalyst during carbazole hydrodenitrogenation correlated with the fractal dimension of the catalyst. It was proposed that this result indicated that the hydrogenation activity was favoured by the irregular surface of the molybdenum crystallite particles (i.e., the grain boundary). 

Furthermore, a series of molybdenum phosphide catalysts supported on alumina was fabricated using temperature-programmed reduction at 823 and 1123 K under hydrogen [57]. The fractal dimension of the catalyst was determined by SAXS and found to correlate linearly with performance in the catalysis of hydrodesulphurization of dibenzothiophene. 

The irregular surface of fractal catalysts affects the accessibility of the active sites. The accessibility of the acid sites of sulfo-cation exchange resins can be improved by forming the active phase as a layer on the interior surface of porous inorganic supports. For example, sulfonated co-polymers of styrene and divinylbenzene were deposited on the interior surface of wide-pore silica gel to use as a catalyst for the synthesis of ethyl t-butyl ether from ethanol and isobutylene [58]. The fractal dimensions of these catalysts were measured by image analysis of SEM micrographs. While it was found that the fractal dimension of the support remained constant at ~2.4, as the polymer content was increased from 0 to 10 wt%, the fractal dimension of the resin phase declined from 2.4 at 0 wt% to ~2.1 at ~4 wt%, then increased thereafter to above 2.6 by 10 wt%. This trend was attributed to the resin initially smoothing the surface by filling in irregularities in the silica and, thereafter, more following the overall topography of the silica. It was found that catalysts with only a partial coverage of resin were the most active in the reaction, and this was attributed to greater access of the acid sites, since thick layers of resin reduced the accessibility of the acid sites. It was suggested that the fractal dimension can characterise the distribution of the active phase.

In situations of diffusion-limited reactions on fractal surfaces, with un(der)-mixed conditions, reaction kinetics different to those of classical equations can develop [59]. The irregular surface of fractals means that, in such circumstances, pockets can develop within the various depth bays in an irregular surface, where the local concentration of reactants is different to elsewhere. Overall, the effect is to make the apparent reaction rate constant in rate equations, using the bulk concentration to vary with time on-stream (or batch time). This effect may result in the decay of the apparent rate constant with time, making it look like the surface had been deactivated in some way (such as by poisoning of active sites) when, in fact, it had not. More complex effects can develop, too, such as the spatial segregation of different species involved in the reaction. The fractal kinetic theory according to which the observed rate is history-dependent can also be applied to adsorption processes [60].

Günnemann et al. [61] found that the fractal kinetics of commercial titania photocatalysts was directly related to the surface roughness of electrodes. They suggested that increasing fractality was due to the formation of agglomerates of smaller powder particles, giving rise to more complex surfaces, while larger particle sizes gave rise to a more even surface.

Jelčić et al. [62] found that the crystal voids of various ZSM zeolites were fractal, with differing fractal dimensions. They found that catalytic peroxide oxidation of phenol over copper catalysts supported on the zeolites was best described by fractional kinetics. In addition, they suggested that the fractality of the structure of the Cu/ZSM-5 catalysts was related to the catalytic activity. 

Peng et al. [63] studied the diffusion-limited catalytic oxidation of formaldehyde over titania-supported platinum pre-reduced at various different temperatures ranging from 300 to 700 °C. The surface-fractal dimensions of the catalysts were measured using nitrogen adsorption and analysis based upon the variable radius of curvature with pressure during capillary condensation. It was found that the fractal model was not followed for relative pressures before the onset of capillary condensation, as indicated by the lower hysteresis closure point. However, it was found that the catalyst with the largest value of the fractal dimension also showed the highest activity at different temperatures. 

Tuning of the fractal dimension can be used to optimize catalyst performance. Since the Knudsen diffusivity depends upon the accessible surface area and size of pores, fractal surface roughness can have a marked impact on diffusion-limited reactions in the Knudsen regime. For example, simulations have demonstrated that purposeful modification of the fractal dimension of the surface of the alumina support of a PtRe catalyst can be used to increase the octane number of the gasoline obtained from an industrial catalytic reformer [38]. The surface-fractal dimension affects the octane number because a higher value indicates a more accessible surface, which contains acid sites that catalyse some of the reforming reactions that increase the octane number [38]. However, an increasing fractal dimension can also be detrimental, since it can also encourage undesired side reactions involving hydrogen (hydrocracking and hydrogenolysis). This occurs because of the relatively higher accessibility of a rougher surface to small hydrogen molecules compared to larger hydrocarbons. Hence, overall, there is an optimum surface-fractal dimension to increase the octane number but not to increase undesired side products.

Dobrescu et al. [64] also attempted to control catalytic properties by modifying the fractal self-similarity of various catalysts. These included oxides LaCoO_3_ and LaFeO_3_ with a perovskite structure obtained under different preparation conditions by thermal decomposition of the precursors with maleic acid, alpha-alanine, urea, and sorbitol. Measurement of the fractal dimensions of these materials was performed using SEM and nitrogen sorption. The gas sorption fractal dimensions of lanthanum ferrites were found to vary with the preparation conditions. Some agreement was found for the fractal dimensions obtained for samples of lanthanum cobaltite doped with varying concentrations of strontium using gas sorption and SEM analysis. Some significant discrepancies between these techniqueswere attributed to the fact that nitrogen sorption only characterises the surface accessible from the exterior, while SEM can characterise all of the internal surface, including that disconnected from the exterior, but can only sample that area to a limited extent. Dobrescu et al. [64] also synthesised and characterised bimetallic nanoparticle catalysts. Fractal analysis of TEM images showed that platinum–copper nanoparticles possessed two regimes of fractal scaling: one for lower length scales and one for larger length scales, separated by a cut-off located at 1.2 nm, which corresponded to the average particle size. This result shows that changes to fractal scaling exponents should be associated with a physical transition in the material, such as from a particle surface to an aggregate envelope surface. Dobrescu et al. [64] also studied rhodium catalysts supported on a range of oxides, namely alumina, titania, and tungsten trioxide. They found a strong correlation between the fractal dimension and the basicity of these catalysts. They also found that adding the metal nanoparticles to the support led to a decrease in the fractal dimension for the composite relative to the support alone. This finding was said to be due to rhodium filling concavities in the surface. However, a discrepancy was observed between the fractal dimensions obtained from gas sorption and TEM. Again, this was attributed to the differences in sampling of the surface by the two experimental techniques. 

Kriston et al. [65] proposed a model for the catalyst layer of a PEM fuel cell that consisted of a self-assembled, self-similar fractal structure rather than randomly distributed spherical agglomerates. The fractal dimension was measured by mercury porosimetry and found to decrease as platinum loading increased. This finding was interpreted to mean that the particles fill out the volume of the catalyst layer less effectively due to a higher degree of agglomeration and screening effects. It was also proposed that there was a relationship between the fractal dimension, the active surface area of the catalyst, and the Thiele modulus.

Besides affecting the mass transport and accessibility of active sites, the fractal roughness also impacts the intrinsic activation energy for surface reactions. One way this effect is mediated is the way in which the surface-fractal roughness affects the transition state by determining the number of nearest neighbouring sites around a particular site on the surface [66]. This, in turn, determines the number of surface sites affected by the activated complex and, therefore, the number of contributions to the energy of activation of the said complex [67]. Indeed, Trypolski et al. [67] found that the activation energy for a CO oxidation reaction increased with an increased surface-fractal dimension for a range of silica-based catalysts. 

The fractal properties of a catalyst surface also affect the intrinsic pre-exponential factor for the Arrhenius dependence of a catalysed rate constant [68]. This effect is mediated by the molecular sieving-like effect produced by a fractally rough surface. This is because the roughness of the surface determines the particular specific surface area as perceived by an activated complex of linear overall size *R*, and, thence, the number of these can be accommodated. According to transition state theory, the pre-exponential factor depends upon the surface concentration of active sites, which is determined by the surface-fractal dimension. Trypolski et al. [68,69] found that the pre-exponential factor for CO oxidation on zirconia decreased significantly with an increasing mass-fractal dimension of the solid.

Where the product of a catalytic reaction is a solid, the fractal dimension can be used to characterise the nature of the product. In combined filtration and catalytic oxidation monolith reactors used to control emissions from diesel engines, it was found that downstream of such reactors, the soot particles had a smoother surface and were denser, as measured by the mass-fractal dimension [70]. The change in the fractal dimension was attributed to a greater reduction in organic matter on the surface of the soot particles and, thus, a reduction in carbon particle sizes, enabling denser packing.

Photocatalysts of increased efficiency were fabricated from rough, high-surface-area, nanocrystalline titania thin films by modification with silver particles [71]. The silver particles were deposited on the titania by dipping in AgNO_3_ solution, then irradiated with UV. Spectroreflectometry demonstrated an increase in optical absorbance, that wouldimprove photocatalytic activity, and which was e attributed to plasmon resonance absorption of the silver clusters. Fractal analysis of atomic force microscopy (AFM) images showed that the silver-doped films exhibited a self-affine scaling character over a wide range of length scales. Falaras et al. [71] attributed this to there being a “chaotic” dynamic deposition process that was very sensitive to the initial conditions. The presence of dynamical chaos is often linked to fractals [71]. It is possible that the texture and morphology of the silver layer deposited on the titania surface might influence the photocatalytic activity. Indeed, Herrmann et al. [72] suggested that the apparent increase in photocatalytic efficiency is due to the increase in the exposed surface due to the textural characteristics of a rough Ag/TiO_2_ layer. Hence, the surface-fractal dimension, which is a measure of surface roughness, might correlate with activity. 

## 5. Computer Simulation and the Design of Fractal Materials for Heterogeneous Catalysis

### 5.1. Catalyst Design via Computer Simulation

Since it has been seen that fractal structures have begun to be synthesized, and that the aforementioned work suggests that fractal properties can improve the performance of catalysts, this suggests that optimal fractal structures might be designed *a priori* for a given reaction and then fabricated to order. With this concept in mind, design of optimal fractal catalyst materials via computer simulation has been attempted.

### 5.2. Catalyst Selectivity

Since there is a much broader distribution in the accessibility of the surface sites of a rough, fractal catalyst structure to reactant molecules diffusing in from the outside, the selectivity of fractal catalysts is different to that of more smooth, Euclidean structures [73]. If there are serial competing reactions, then the potential deep pockets or ‘fjords’ in the rough surface of a catalyst not present in smoother Euclidean catalysts means molecules are likely to get trapped within and spend more time in the proximity of the catalyst. Hence, reactant or product molecules are more prone to further reaction in fractal catalysts. In diffusion-limited systems, more rapid chemical reactions are most likely to occur on the more exposed outer pinnacles of an irregular fractal surface, whereas slower reactions occur deeper into the ‘fjords’ within the structure, as the reactants have time to penetrate therein. Hence, there are very different chances of the products of these two reactions escaping from their particular origin region before impacting other incoming molecules and/or more catalytic surface sites, leading to different chances of further reaction. The molecular sieving effect, as depicted schematically in Figure 3, can also affect reaction selectivity, as already described above [38]. In order to consider the impact of fractal geometry on chemical reaction selectivity, a number of simulation studies have been conducted on various fractal models, as summarized in Table 1. The diffusion-limited aggregate (DLA) model is frequently used for metal-particle and carbon aggregates.

### 5.3. Optimisation of Mass Transport Properties

**Figure 8:**
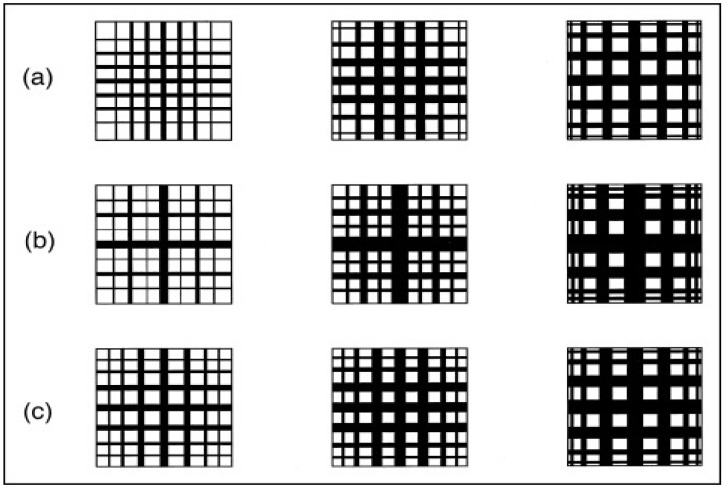
(**a**–**c**) Optimal geometry of square networks with 7 × 7 pores. Pores are represented as black, and the microporous catalytic support is indicated by white. Reprinted with permission from Ref. [79], 2004, John Wiley and Sons.

Gheorghiu and Coppens [79] compared the catalytic performance of self-similar, fractal-like pore hierarchies with that of more uniform pore structures, as depicted in Figure 8. They constructed (strictly) pre-fractals with up to five generations of the recursive fractal-generating process. Overall, these workers found that a fractal structure provided some advantage over a more uniform structure only in the Knudsen and transitional diffusion regimes. This was because, when the mass transport resistance of pores depended upon their size, the fractal structure provided greater accessibility to the smaller pores via the hierarchical tree of larger pores. However, the effectiveness factor for fractal and uniform structures was similar, even in the Knudsen regime. Instead, the advantage of fractal structures arose from the higher activity per unit of catalyst mass in a given volume. For example, the activity was up to ~40% greater for the fractal structure compared with the more uniform counterpart.

**Figure 9:**
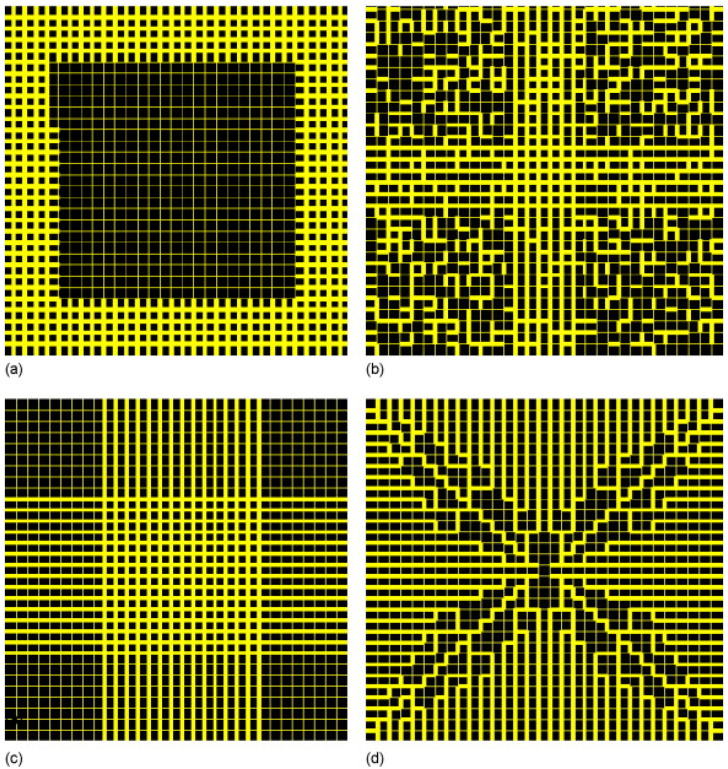
Illustrative re-assembled archetypal pore networks: (**a**) minimum shielding; (**b**) partial cruciform; (**c**) fully cruciform; (**d**) fractal tree. Reprinted with permission from Ref. [80], 2007, Elsevier.

Prachayawarakorn and Mann [80] also compared the performance of a fractal tree-like structure with both more uniform structures and a minimized pore-shielding structure, as shown in Figure 9. They found that the network’s effectiveness was maintained to a higher Thiele modulus for the fractal tree compared with every other tested structure, except the minimum shielding structure. However, given that only a limited range of pore sizes was used in the construction of the models, it is not clear whether the fractal tree arrangement might perform relatively better if a wider pore size range were used, as in the pore-shielding model, the relatively much larger active surface area of smaller pores may have been buried deep within the heart of the minimum pore-shielding structure.

Fractal structures can be more resistant to deactivation and more efficiently re-activated. El-Nafaty and Mann [81] showed that a pore network design for fluidized-catalytic-cracking catalyst particles consisting of a pre-fractal structure where, on average, the small pore-to-small pore links were minimized, whilst, at the same time retaining a high large pore-to-large pore linkage, gave rise to the quickest coke conversion with time during coke burn-off. A fractal tree-like structure might be expected to be more durable in terms of resisting deactivation by coke deposition than a more disordered random pore catalyst. This is because the larger (trunk-like) pores provide ready access from the exterior to progressively smaller (branch- and twig-like) pores, which may not be present in random systems where small pores can block access to larger pores. Hence, a catalyst with a fractal tree-like pore structure might be expected to have a longer lifetime before deactivation due to pore-blocking with coke.

## 6. Outlook

The initial wave of interest in the potential of fractal theory to aid in understanding and designing new catalysts followed the publication of the books by Mandelbrot [82] and then Avnir [1]. As can be seen from the above discussion, much work on the utilisation of fractal theory and concepts in catalysis has continued since. Recently, there has been a new wave of interest in the use of fractal concepts for the structural characterisation of natural materials, especially rocks and minerals and, in particular, shales that are gas reservoirs or carbon storage seal caprocks [83]. This is because, as mentioned above, fractal theory provides a way to characterise otherwise inscrutable, highly complex, heterogeneous systems to provide parameters to correlate and predict physical processes like mass transport, adsorption, and reaction occurring within them [84]. Fractal-based modelling approaches do not suffer from the drawback of the resolution vs. field-of-view trade-off necessary with application of imaging-based modelling to rocks. The application of such fractals to natural materials, and waste derived from them will also be increasingly relevant to catalysis with the expanding use of such materials as a source of cheap catalysts needed for processes involving low-margin products and/or systems where catalysts rapidly deactivate and cannot be regenerated or recovered. 

Such materials include minerals located in situ within reservoir rocks, which act as natural catalysts for thermochemical processing of fluids downhole, such as for upgrading heavy oil [85],and downhole production of hydrogen from methane [86]. Such minerals include kaolinite, chlorite, and albite. Core drilling enables samples of such rocks to be brought into the lab for fractal-based characterisation and testing. Other materials include waste such as so-called ‘red mud’ (RM) [87], also known as bauxite residue, which is composed of catalytically active metal oxides. RM consists of chemical phases of Bayer sodalite (Na_8_Al_6_Si_6_(OH)_24_CO_3_), unreacted boehmite (gamma-AlO(OH)), hematite (Fe_2_O_3_), Al_2_O_3_, quartz (SiO_2_), and TiO_2_ recovered from the Bayer process during alumina production. Similar mineral waste also arise in secondary phases linked to mining and geothermal energy production activities, and industrial use of these as catalysts helps to make the primary process cost-effective. Further waste materials are also starting to be used as catalyst supports, including metal slags such as Ni slags. Fractal-based characterisations have been increasingly applied to such materials due to their inherent complexity [88]. 

Increasing sophistication of material syntheses will enable the higher levels of fine control needed to produce ever more complex fractal materials, both in the lab and in plants. It will also enable the degree of durability of finely structured fractal catalyst materials to be improved in the face of the harsh reaction conditions required for some catalytic processes.

Metal-oxide semiconductors such as titania can exhibit strong dielectric Mie resonances that enhance the heterogenous catalytic activity of several reactions, such as coupling reactions [89,90]. It is possible that a fractal geometry might further enhance these resonance effects by providing additional surface roughness and hierarchical porosity. Since dielectric Mie resonances depend upon the size and shape of the catalytic nanoparticles, the particular fractal dimension of a catalyst surface may influence the resonance frequencies and local electric field enhancement in these systems [91,92]. The possibility of these effects should be investigated in the future.

## 7. Conclusions

- The nature of fractal models and materials as governed by explicit mathematical scaling laws rather than the more ‘black-box’ relations of artificial intelligence models like neural networks, means the fractal models can be interrogated more easily and that extrapolation to, say, different length scales beyond those currently considered is also feasible.
- Studies of various different types of catalysts have shown that discrepancies can arise between the measured fractal dimensions obtained by different techniques. For discrepancies between microscopy and more indirect methods like gas sorption, this is commonly due to big differences in the nature and degree of sampling of the structure of the material. The discrepancies between the surface-fractal dimensions obtained from gas sorption and SAXS often arise due to the properties of the real physical system not matching the assumptions made in the model of adsorption used to analyse these data, such as surface chemical homogeneity. The size of these discrepancies can be as large as 0.3–0.5 for surface-fractal dimensions ranging from 2 to 3.
- It has been seen that a variety of fractal materials can now be fabricated with varying degrees of control over the fractal dimension, and these new materials can also be used as catalysts themselves or supports. The range of fractal dimensions that can be obtained covers the full range possible, such as from 2 to 3 for surface fractals.
- The fractal dimension has been found to be a key descriptor of the structure of catalysts that is physically relatable to the observed performance of such materials, providing a guide for understanding and control.
- The further possibility of controlled synthesis offers the potential to design the optimum fractal materials for a given catalytic system. Attempts have been made to design such materials in silico via computer simulation of the physico-chemcial processes involved in catalytic reactions in fractal models.

## Figures and Tables

**Table 1:** Computer simulation studies of the impact of fractal geometry on catalyst selectivity. (A, B, C, relevant molecules; S, surface site; * surface-adsorbed).

Fractal Model	Reactions	Impact of Fractality and/or the Fractal Dimension	Reference
2D DLA	(i) A→B (ii) A + A→C	The dependence of the catalyst selectivity (C/B ratio) on the first-order rate constant exhibits a non-classical power-law behaviour.	[74]
2D DLA	(i) A + S→A* (ii) A*→B (iii) A + A*→C	Varying the reaction-step probabilities can lead to the existence of a maximum or minimum in selectivity.	[75]
2D DLA	(i) A + S→A* (ii) A*→B (iii) A + A*→C	The reaction probability distribution of the Eley–Rideal reaction mechanism was shown to have multi-fractal characteristics. The reaction probability distribution for model sites becomes more heterogeneous as DLA size increases.	[76,77]
2D DLA	(i) A + S→A* (ii) A*→B (iii) A + A*→C	The macroscopic average selectivity of a fractal catalyst behaves differently to a smooth catalyst. The dependence of the catalyst selectivity (C/B ratio) on the rate constant exhibits a non-classical power-law behaviour. Selectivity increases as the fractal dimension of the DLA increases.	[78]
3D CCA & 3D Menger Sponge	(i) A + S→A* (ii) A*→B (iii) A + A*→C	A general trend of increasing selectivity with a decreasing mass fractal dimension was observed. However, the rate of change of selectivity with the mass-fractal dimension decreases with a decreasing fractal dimension, particularly for mass-fractal dimensions less than ∼2.95.	[73]

## Data Availability

Not applicable.
